# Validation of the Marijuana Effect Expectancies Questionnaire (MEEQ) in a Non-Clinical French-Speaking Adolescent Sample

**DOI:** 10.5334/pb.263

**Published:** 2016-02-02

**Authors:** Emilie Schmits, Etienne Quertemont, Eric Guillem, Cécile Mathys

**Affiliations:** 1 Département Psychologie, Cognition et Comportement, Université de Liège, Belgium; 2 Fonds National de la Recherche Scientifique, Belgium; 3 Groupe Hospitalier Lariboisière-Fernand Widal (APHP), Paris, France; 4 Département de Criminologie, Université de Liège, Belgium

**Keywords:** Marijuana Effect Expectancies Questionnaire, cannabis, expectancies, teenagers, psychometric properties

## Abstract

Teenagers commonly use cannabis. Expectancies related to the effects of cannabis play an important role in its consumption and are frequently measured with the Marijuana Effect Expectancies Questionnaire (MEEQ). This study aims to assess the psychometric properties (factor structure, internal consistency reliability, criterion validity) of the French MEEQ. A sample of 1,343 non-clinical teenagers (14–18 years) were recruited to answer a self-report questionnaire; 877 of them responded twice (one-year interval). A four-factor structure was obtained: Cognitive Impairment and Negative, Relaxation and Social Facilitation, Perceptual Enhancement and Craving and Negative Behavioral Effect Expectancies. It is concluded that the French MEEQ constitutes an appropriate tool to measure cannabis effect expectancies among adolescents.

Cannabis is a drug commonly used by teenagers in occidental countries. Although its lifetime use prevalence does not appear to have increased in recent years, 23% of Belgian youths (15–16 years) use it, with an average use level of 30% in Europe and 35% in the United States (EMCDDA, 2012). Cannabis use has negative consequences, especially on physical and mental health (Degenhardt et al., 2012; Patton et al., 2002; Zvolensky, Bonn-Miller, Leyro, Johnson, & Bernstein, 2010), and leads to daily problems such as social impairments (Zvolensky et al., 2010). Younger users are more likely to experience the negative psychosocial effects of regular use (Fergusson, Horwood, & Swain-Campbell, 2002; Lynskey et al., 2003) and risky behaviors are more common in youths (e.g. aggressiveness, premature sexual behavior) (Kokkevi, Gabhainn, & Spyropoulou, 2006). Early assessment of cannabis use seems relevant, especially for timely prevention and intervention. Therefore, adolescence is an essential period to investigate cannabis consumption.

Expectancies related to the effects of cannabis use play an important role in its consumption in young adults (Beraha, Cousijn, Hermanides, Goudriaan, & Wiers, 2013; Gaher & Simons, 2007; Galen & Henderson, 1999; Simons & Arens, 2007) and adolescents (Kristjansson, Agrawal, Lynskey, & Chassin, 2012; Neighbors, Geisner & Lee, 2008; Skenderian, Siegel, Crano, Alvaro, & Lac, 2008). Indeed, as part of the normal learning process, representations and expectancies exist even before the first consumption of a substance, and these cognitions could be considered as potential components for prevention strategies (Wiers et al., 2003). Teenagers who have never smoked cannabis have a more negative perception of it than those who have already tried it (Alfonso & Dunn, 2007; Linkovich-Kyle & Dunn, 2001; Schmits, Mathys, & Quertemont, 2015, in press). Furthermore, more frequent cannabis use was observed in consumers with positive expectancies (e.g. euphoric effects), whereas less frequent use was associated with negative expectancies (e.g. deleterious effects on health or behavioral control; Simons & Arens, 2007). The severity of use also can be predicted by global negative effect expectancies (Hayaki et al., 2010). Cannabis effect expectancies could therefore be used as a tool for prevention or intervention in the context of cannabis use and abuse.

Several tools have been created to assess cannabis effect expectancies, including the Cannabis Expectancy Questionnaire (CEQ) (Young & Kavanagh, 1997), the Adolescent Cannabis Expectancies Questionnaires (AECQ) (Willner, 2001) and the Nicotine and Marijuana Interaction Expectancy questionnaire (NAMIE) (Ramo, Liu, & Prochaska, 2013). However, the Marijuana Effect Expectancies Questionnaire (MEEQ) (Schafer & Brown, 1991) is one of the most frequently used (Buckner & Schmidt, 2008, 2009; Hayaki et al., 2010; Neighbors et al., 2008; Perez, Ariza, Sanchez-Martinez, & Nebot, 2010). The MEEQ is a 48-item list of expectations about cannabis use. Its major advantage is that it can be completed by people with and without a history of cannabis consumption. Indeed, the instructions of the scale explicitly ask non-user participants to project themselves into cannabis consumption and to imagine the effects they could expect if they use the substance. Although cannabis effect expectancies and cannabis use motives overlap to some extent and are clearly related, it is important to make a distinction between them. Expectancies do not necessarily lead to cannabis use (i.e., nonusers also have cannabis effect expectancies), whereas subjective motives are usually studied a posteriori in cannabis users (Bonn-Miller & Zvolensky, 2009; Hecimovic, Barrett, Darredeau & Stewart, 2013). In the MEEQ, respondents have to specify the degree to which they expect the occurrence of effects as a result of cannabis consumption on a Likert scale ranging from 1 (*strongly disagree*) to 5 (*strongly agree*). The original English MEEQ was validated in nonclinical and clinical samples of young adults (Galen & Henderson, 1999; Schafer & Brown, 1991) and in clinical and community samples of adolescents (Aarons, Brown, Stice, & Coe, 2001). A six-factor structure was identified (48 items): Cognitive/Behavioral Impairment, Relaxation/Tension Reduction, Social/Sexual Facilitation, Perceptual/Cognitive Enhancement, Global Negative Effects, and Craving/Physical Effects (Aarons et al., 2001; Schafer & Brown, 1991). Moreover, a very brief version (6 items) of the MEEQ (MEEQ-B) was validated in a clinical sample of adolescents (Torrealday et al., 2008). This scale is mainly used in scientific studies. However, its use in the clinical context might also be considered for prevention, risk reduction or therapeutic intervention.

To date, there is no fully validated French version of the MEEQ for adolescents. Guillem et al. (2011) published a French translation of the questionnaire with good psychometric properties. The authors assessed the psychometric properties of the scale in a clinical sample of adults and reported an exploratory four-factor structure (31 items): (1) Cognitive Impairment and Negative Effects (e.g. « If I have smoked cannabis, it is harder to remember things »), assessing the cognitive modifications (e.g. lower concentration, slowed thoughts, loss of control) expected after consumption; (2) Relaxation and Social Facilitation (e.g. « I found a sense of relaxation by smoking cannabis »), probing into the anxiolytic (e.g. relaxation, relief) and socializing (e.g. disinhibition, fun) effect of use; (3) Perceptual Enhancement and Craving (e.g. « Smoking cannabis increases my immediate desire for things »), assessing the improvements in creativity and in the interest or desire for things expected after cannabis use; and (4) Negative Behavioral Effects (e.g. « Cannabis can make me angry and makes me potentially violent »), probing into the negative behaviors or sensations expected with cannabis use. Some of the original 48 items were not included due to their low factor loadings (less than 0.40), reducing the number of items to 31. Such a reduction was reported to improve the clarity of the model and its clinical meaningfulness.

Participants in the study conducted by Guillem et al. (2011) were adult cannabis users, psychiatric inpatients and control subjects. Validating the French MEEQ and its factor structure in a non-specific sample of adolescents is therefore important. Guillem et al. (2011) themselves indicate that future research is necessary to confirm the factor analysis in a representative sample from the general population. It would be especially useful to compare the two theoretical factor structures published in the literature (six factors vs. four factors) to see which of them shows the best fit in a general population sample.

The main goal of the present study was therefore to test those two pre-existing theoretical models in a large general population sample of adolescents in order to propose a validated French version of the MEEQ adapted for use in adolescents. In this context, the specific aims of the present study were: (1) to compare the two theoretical factor structures of the MEEQ (six factors with 48 items vs. four factors with 31 items) on several fitting indexes: CFI (Comparative Fit Index), RMSEA (Root Mean Square Error of Approximation), BIC (Bayesian Information Criterion), AIC (Adjusted Information Criterion) and χ^2^/df; (2) to assess the psychometric properties of the best of these models with descriptive and internal consistence reliability (Cronbach’s alpha) statistics; and (3) to explore the criterion validity by testing the concurrent validity (association between factors and current cannabis use) and the predictive validity (predictive value of the factors for future cannabis use in a longitudinal perspective).

## Method

### Participants

A sample of 1,343 (49.59% female) teenagers were recruited from 11 high schools (Grade 10) representing all educational networks in a French-speaking area of Belgium (Liège) (*M* = 15.70 years, *range* = 14–18, *SD* = 0.88). Only native French speakers completed the questionnaire. Initial phone contact was made with a large number of randomly selected schools (52 schools) in order to obtain at least 10 schools in the final sample (finally 11 schools were included in the study). The main reasons for refusal were the high number of requests to participate in scientific studies, as well as other commitments in long term studies. The reported reasons for refusal were independent of the purpose of the study. This sample was drawn from a recent longitudinal investigation on adolescent substance use (Schmits, Mathys, & Quertemont, 2015), from 2012 to 2014. We used wave 1 (January–April 2012) and wave 2 (January–April 2013) for the present study, following the students from Grade 10 to Grade 11.

Of the 1,343 participants in wave 1, 1,017 (75.72%) had never used cannabis and were classified as “non-users”; 326 (24.27%) had used this substance and were classified as “cannabis users” (according to their answer on the item: “Have you already used cannabis? Yes or No”).

### Procedure

Data were obtained with a self-report questionnaire collectively in class, without the participation of teachers. Participants did not receive any compensation for participation. The study protocol was approved by the University’s Institutional Review Board. Informed consent was obtained from parents and students prior to data collection. In case of refusal, a subject would receive a questionnaire and return it blank (fewer than 10 students and parents did so). A confidential identification code was created for each participant and was used for all identifying information.

### Measures

***Marijuana Use Form (MUF)*.** The MUF is a self-report measure used to assess cannabis use (Buckner, Bonn-Miller, Zvolensky, & Schmidt, 2007; Buckner, Crosby, Silgado, Wonderlicht, & Schmidt, 2012; Buckner, Heimberg & Schmidt, 2011; Buckner, Silgado & Schmidt, 2011). Participants report whether they have ever used cannabis, the date of last use, and the usual frequency of use (lifetime, past month, and past week).

***Marijuana Effect Expectancies Questionnaire (MEEQ)*.** As described above, the French version of the instrument (Guillem et al., 2011), with the 48 original items (Schafer & Brown, 1991), was used. The full French scale is shown in Appendix A. This tool assess different expectancies about the effects of cannabis, throughout a Likert scale ranging from 1 (*strongly disagree*) to 5 (*strongly agree*). A high score on a specific item (for example relaxation and social facilitation) means that the participant expressed a high level of this expectancy. The questionnaire was designed in a way that allows completion by both cannabis users and adolescents who have never used cannabis. Note that the scores of MEEQ items 27, 32 and 36 must be reversed before analysis as instructed by the questionnaire manual (negatively worded items). For the original six-factor structure (Schafer & Brown, 1991), the factors were defined as follows: Cognitive and Behavioral Impairments (items 14, 15, 17, 18, 21, 33, 36, 40, 43, 46), Relaxation and Tension Reduction (items 4, 5, 6, 7, 10, 26, 42, 44), Social and Sexual Facilitation (items 8, 12, 13, 24, 27, 31, 32, 45, 47), Perceptual and Cognitive Enhancement (items 1, 9, 16, 19, 22, 26, 29, 37), Global Negative Effects (items 11, 23, 25, 28, 30, 34, 35, 38, 48), Craving and Physical Effects (items 2, 3, 20, 33, 39, 41). For the French four-factor structure (Guillem et al., 2011), the factors were defined as follows: Cognitive Impairment and Negative Effects (items 14, 15, 17, 18, 21, 25, 28, 33, 35, 40, 43, 46), Relaxation and Social Facilitation (items 4, 5, 10, 12, 19, 24, 44, 45), Perceptual Enhancement and Craving (items 1, 3, 8, 9, 16, 22), Negative Behavioral Effects (items 11, 23, 31, 34, 48).

### Data Analysis

The six-factor structure (the original 48 items) validated in the English version of the MEEQ was first compared with the four-factor structure (31 items) proposed by Guillem et al. (2011). Such a validation was carried out on the data from wave 1 of the study using Mplus. Given that statistical units were clustered in classes and schools (non-normal and non-independent observations), robust maximum likelihood estimation (MLR) was used (Muthen & Muthen, 2010). As recommended by Hu & Bentler (1999), the Confirmatory Factor Analyses (CFA) model fit was tested. As a minimum of three indicators per factor is usually required (Velicer & Fava, 1998), we used the Comparative Fit Index (CFI), Root Mean Square Error of Approximation (RMSEA), and χ^2^/df ratio, with values respectively >0.95, <0.08, and <3.00 to indicate adequate fit (Bentler, 1990; Bentler & Bonett, 1980; Bollen, 1989; Browne & Cudeck, 1989; MacCallum, Browne & Sugawara, 1996). To compare CFA models with a different number of factors and select the more parsimonious model (four-factor structure model compared to six-factor model), we also used the Bayesian Information Criterion (BIC; Schwartz, 1978), Sample-Size Adjusted Bayesian Information Criterion (ABIC; Sclove, 1987) and Sample-Size Adjusted Information Criterion (AIC, Sclove, 1987), with a better fit indicated by smaller values. Adequacy of factor loadings and Cronbach’s alpha coefficients were examined, factor loadings >0.40 being considered as acceptable (Ford, MacCallum & Tait, 1986; Hair, Anderson, Tatham, & Black, 1998). For the best model, we generated descriptive statistics, correlations among these factors, and internal consistency (SPSS 18 software). These data are also available for the alternative model in Appendix B. Finally, we assessed the criterion validity of the best model in order to test whether these factors are able to predict cannabis use: concurrent validity related to cannabis use in wave 1 (using the dichotomous variable: “never used cannabis” versus “already used cannabis” and the usual frequency of cannabis use for adolescents identified as users; Buckner et al., 2007); predictive validity by examining the relation between the MEEQ scores in wave 1 and the consumption of cannabis in wave 2 (from the dichotomous variable “never used cannabis” versus “already used cannabis,” three profiles of cannabis users were computed: “non-users,” “users at baseline,” and “new users”) (see results section for more details). Finally, the relationship between the MEEQ expectancy scores in wave 1 and the frequency of cannabis use in wave 2 was also assessed to test the predictive validity. Zero-order correlations were computed (SPSS 18 software) for concurrent validity (variables in wave 1), as well as multinomial logistic regressions and multiple regressions for predictive aspects (wave 1 and wave 2). The same data for the alternative model are also available in Appendix B.

Statistical significance was set at p < 0.05. For multiple regressions, the bootstrapping method (10,000 bootstrap samples) was used. This method is a non-parametric approach to effect-size estimation and hypothesis testing that is not based on large-sample theory and, therefore, circumvents the power problem due to asymmetries (Shrout & Bolger, 2002).

In summary, the following analyses were conducted: Confirmatory Factors Analysis to test the factor structure, elementary statistics (means, standard deviations and Cronbach’s alphas) to assess descriptive statistics and internal consistency reliability, zero-order and Spearman correlations to determine the concurrent validity, multinomial logistic regression as well as multiple regression analyses to test predictive validity.

## Results

### Factor Structure of the MEEQ

Table 1 shows the model fitting statistics of both the six-factor structure (48 items) and the four-factor structure (31 items) that were computed on wave 1 data. The four-factor structure was consistently superior and more parsimonious with smaller BIC, ABIC and AIC indices. Moreover, the CFI, RMSEA, and χ^2^/df ratio for the four-factor model were equal to or better than those of the six-factor structure.

**Table 1:** Fit Indices for the CFA Models.

Model	CFI	RMSEA	90% CI	BIC	ABIC	AIC	χ^2^/df

1. Four-factor	0.83	0.05	0.04–0.05	153240.24	152916.25	152720.96	3.70
2. Six-factor	0.74	0.05	0.04–0.05	227757.83	227246.43	226938.20	3.49

*Note*. CFI = Comparative Fit Index, RMSEA = Root Mean Square Error of Approximation, CI = Confidence Interval, BIC = Bayesian Information Criterion, AIC = Adjusted Information Criterion.

Given that the four-factor structure shows a better fit than the six-factor structure, the four-factor model was more deeply analyzed below (similar results for the six-factor model are available for comparison in Appendix B). Structural analyses of the four separate factors revealed that the model had values of less than 3.00 for the χ^2^/df ratio and each factor presented the ideal fit level of .95 for CFI and .06 for RMSEA. Moreover, the upper limit of the 90% CI for the RMSEA was inside the boundary (i.e. <.08). Specifically, statistics for each factor are: χ^*2*^ (53) = 92.741, CFI = 0.97, RMSEA = 0.02 for the factor « Cognitive Impairment and Negative Effects »; χ^2^ (18) = 22.60, CFI = 0.99, RMSEA = 0.01 for the factor « Relaxation and Social Facilitation »; χ^2^ (9) = 23.39, CFI = 0.97, RMSEA = 0.04 for the factor « Perception Enhancement and Craving »; χ^2^ (5) = 14.36, CFI = 0.98, RMSEA = 0.04 for the factor « Negative Behavioral Effects ». Except for a few items (items 8, 22, 28, 31, 35, 45, 46 and 48), all the indicators showed a factor loading greater than .40, meeting the traditional cut-off point for factor loadings (Ford, MacCallum, & Tait, 1986; Hair et al., 1998). The items listed above showed a lower standardized factor loading, suggesting that they are weaker indicators of the factors. As they were statistically significant and were included in the previously published model, we retained these items in the model.

### Descriptive Statistics and Internal Consistency Reliability of the Four Factors of the MEEQ

Descriptive statistics and internal consistency reliability for the whole sample (Wave 1) are given in Table 2 (see Appendix B for the six-factor model). According to the rules proposed by Nunnally (1978), two factors have good reliability (Cognitive Impairment and Negative Effects, α = 0.83; Relaxation and Social Facilitation, α = 0.81). The two other factors have Cronbach’s alphas below 0.70 (Perceptual Enhancement and Craving, α = 0.63; Negative Behavioral Effects, α = 0.60), which would be considered as satisfactory according to Hair et al. (2009).

**Table 2:** Descriptive Statistics and Cronbach’s Alphas for Each of the Four Factors.

	Items	Min	Max	*M*	*SD*	*M_Item_*	α

MEEQ							
Cognitive Impairment and Negative Effects	12	12.00	57.00	38.28	7.00	3.19	0.83
Relaxation and Social Facilitation	8	8.00	40.00	26.92	5.39	3.36	0.81
Perceptual Enhancement and Craving	6	6.00	30.00	18.32	3.59	3.05	0.63
Negative Behavioral Effects	5	5.00	25.00	15.26	3.40	3.05	0.60

*Note*. Min = Minimum, Max = Maximum, M = Mean of the factor, SD = Standard Deviation, Mitem = Mean of the factor/the number of items, α = Cronbach’s alpha.

### Criterion Validity between the Four MEEQ Expectancies and Cannabis Use

***Concurrent validity*.** Zero-order correlations and Spearman correlations were computed among the four factors of the MEEQ and the cannabis variables at wave 1 (Table 3; see Appendix B for the six-factor structure). Lifetime cannabis use was significantly correlated with all these variables, positively with Relaxation and Social Facilitation and with Perceptual Enhancement and Craving expectancies, and negatively with Cognitive Impairment and Negative Effects and with Negative Behavioral Effect expectancies. Among users, the usual frequency of cannabis consumption was positively correlated with Perceptual Enhancement and Craving expectancies and with Relaxation and Social Facilitation and negatively with Negative Behavioral Effects expectancies. Note that the significance of the results is similar when sex and age are controlled, except the correlation between frequency of use and cognitive impairment and negative effects expectancies (r = .12, p = .03) which became significant because of suppression.

**Table 3:** Zero-Order and Spearman Correlations among MEEQ Factors and Cannabis Use Variables.

	Whole sample (n = 1343)					Users (n = 325)
	
	1.	2.	3.	4.	5.	6.

1. Lifetime cannabis	1.00					
2. Cogn. Impairment	–0.30**	1.00				
3. Relaxation	0.16**	0.34**	1.00			
4. Percept. enhance.	0.11**	0.30**	0.63**	1.00		
5. Negative behavior	–0.50**	0.62**	0.16**	0.16**	1.00	
6. Frequency of use		0.09	0.33**	0.32**	–0.22**	1.00

*Note*. Cannabis use (no = 0, yes = 1); Users = subjects who had used cannabis; Cogn. impairment = cognitive impairment and negative effects expectancies; Relaxation = relaxation and social facilitation expectancies; Percept. enhance. = perceptual enhancement and craving expectancies; Negative behavior = negative behavioral effects expectancies. *p < 0.05, **p < 0.001

***Predictive validity*.** Among the 877 participants at both time 1 (T1) and time 2 (T2) (49.94% female; *M* = 15.61, *SD* = 0.81), 693 (79.02%) had never used cannabis at the baseline, whereas 184 (20.98%) had already tried cannabis. At T2, 580 (66.13%) reported never having used cannabis, against 297 (33.87%) who had already tried it. During the follow-up period of one year, 113 students (12.89%) therefore used cannabis for the first time and were classified as new users. Among the 877 students, then, 580 (66.13%) were “non-users,” 184 (20.98%) were “users at the baseline” and 113 (12.89%) were “new users” (Table 4).

**Table 4:** Percentage of cannabis use at T1 and T2.

n (%)	T1	T2

**Non-users**	693 (79.02%)	580 (66.13%)
**Users at baseline/Users**	184 (20.98%)	297 (33.87%)
**New users between T1 and T2**	113 (12.89%)	

A multinomial logistic regression with multiple predictors was conducted (Table 5; see Appendix B for the six-factor model) to examine the predictive relations between MEQQ expectancies at wave 1 and profiles of cannabis users at wave 2 (dependent variable; “non-users,” “users at baseline,” and “new users, with “non-users” defined as the reference category). This model shows adequate effects: R^2^ = 0.30 (Cox and Snell), 0.36 (Nagelkerke); Model χ^2^ (8) = 307.86, p = 0.00. Three of the four cannabis effect expectancies at wave 1 significantly predicted whether adolescents were “users at baseline” or “non-users”: Relaxation and Social Facilitation; Perceptual Enhancement and Craving; and Negative Behavioral Effects. Cognitive Impairment and Negative Effects expectancies at wave 1 were not related to being a “user at baseline” or “non-users” at wave 2. Regarding the profile of “new users” versus “non-users,” all expectancies were significant predictors: Cognitive Impairment and Negative Effects; Relaxation and Social Facilitation; Perceptual Enhancement and Craving; and Negative Behavioral Effects. In short, adolescents with negative expectancies (especially negative behavioral effects) were less likely to try cannabis in the follow-up period. Those with positive expectancies (relaxation and social facilitation, perceptual enhancement and craving effects) were more likely to be “users at baseline” or to become new cannabis users during the follow-up period.

**Table 5:** Predictive Validity Results of the Multinomial Logistic Regressions Analyses of Profiles of Cannabis Users.

	b	SE b	Wald	OR (95% CI)

Users at baseline vs. Non-users				
Constant	–3.17**	0.97	10.74	
Cognitive Impairment Expectancies	0.03	0.02	1.30	1.03 (0.98–1.07)
Relaxation Expectancies	0.07*	0.03	5.80	1.07 (1.01–1.14)
Perceptual Enhancement Expectancies	0.11*	0.04	6.43	1.11 (1.02–1.21)
Negative Behavior Expectancies	–0.22**	0.05	19.99	0.81 (0.73–0.89)
New users vs. Non-users				
Constant	1.85*	0.70	7.02	
Cognitive Impairment Expectancies	–0.05*	0.02	6.06	0.95 (0.91–0.99)
Relaxation Expectancies	0.13**	0.03	20.43	1.13 (1.07–1.20)
Perceptual Enhancement Expectancies	0.17**	0.04	17.30	1.18 (1.09–1.28)
Negative Behavior Expectancies	–0.50**	0.05	111.11	0.60 (0.55–0.66)

*Note*. Non-users was the reference category. OR = Odds Ratio. *p<0.05, **p<0.001.

Table 6 shows the results of the multiple regression analysis that was conducted to predict the usual frequency of cannabis use among users at wave 2 (“users at the baseline” plus “new users,” n = 297) from the four cannabis expectancies at wave 1 (see Appendix B for the six-factor model). This model accounted for 10% of the total variance in usual frequency of cannabis consumption: R^2^ = 0.10; adjusted R^2^ = 0.09; F (4, 277) = 48.52, p = 0.00. Expectancies that were significant predictors of usual frequency of cannabis consumption include positive associations with perceptual enhancement and craving and a negative link with behavioral effects.

**Table 6:** Predictive Validity – Results of the Multiple Regression Analyses of Usual Frequency of Cannabis Use.

	b	SE b	ß

MEEQ			
Constant	2.68 (1.53, 3.80)	0.59	
Cognitive Impairment and Negative Effects	–0.01 (–0.33, 0.20)	0.14	–0.04
Relaxation and Social Facilitation	0.01 (–0.22, 0.40)	0.18	0.04
Perceptual Enhancement and Craving	0.05 (–0.01, 0.12)*	0.28	0.15
Negative Behavioral Effects	–0.11 (–0.16, –0.05)**	0.26	–0.28

*Note*. Multiple regression with 95% bias corrected and accelerated confidence intervals reported in parentheses. Confidence intervals and standard errors based on 1000 bootstrap samples. *p < 0.05, **p < 0.001.

## Discussion

This study assessed the structural and psychometric properties of a French version of the MEEQ in a large non-clinical adolescent sample and compared the four-factor model proposed by Guillem et al.’s (2011) with the original six-factor model of the English version of the MEEQ (Aarons et al. 2001; Schafer & Brown, 1991). The French version of the MEEQ demonstrated an adequate four-factor structure and good psychometric properties in adolescents.

Among the two tested models, the fit indices revealed that the more adequate and parsimonious model was the four-factor structure. However, the global CFI value for the four-factor structure was below 0.90 (see Table 1). CFI is an incremental measure of fit that may not be particularly informative if the RMSEA of the null model is less than 0.158, as it would generate an overly small value of fit. A RMSEA of 0.05 and a Tucker-Lewis Index (TLI) of less than 0.90 would imply that the RMSEA of the null model is 0.158 (Barrett, 2007; Kenny, 2014). As the RMSEA of our data is 0.05 and the TLI is 0.81, one cannot rule out the possibility that the low CFIs are the result of this phenomenon. Another point to discuss is that some items showed a loading < 0.40, contributing weakly to their respective factors. Following Wang & Wang (2012), we did not exclude these items because all items loaded significantly on their factors and all fits were good. Keeping all items in the model therefore allows to be consistent with the use of the MEEQ in previously published studies (e.g. Guillem et al. 2011 for the French version). Furthermore, suppressing these items did not significantly improve the adequacy of fits (data not shown).

Consistent with Guillem et al. (2011), two factors (Cognitive Impairment and Negative Effects and Relaxation and Social Facilitation) of the four-factor model demonstrated good Cronbach’s alphas, higher than the acceptable threshold of .70 suggested by Nunnally (1978). The other two factors (Perception Enhancement and Craving and Negative Behavioral Effects) showed lower values. However, Hair et al. (2009) argued that values between 0.60 and 0.70 define the lower limit of acceptability. Consistently with previous studies, internal consistency could therefore be considered as satisfactory (Aarons et al., 2001; Connor, Gullo, Feeney, & Young, 2011; Ramo, Liu, & Prochaska, 2013; Schafer & Brown, 1991; Torrealday et al., 2008).

With respect to concurrent validity, and consistent with previous studies (Alfonso & Dunn, 2007; Hayaki et al., 2010; Linkovich-Kyle & Dunn, 2001; Simons & Arens, 2007), significant correlations were found between lifetime cannabis consumption and effects expectancies. Overall, the more adolescents report positive expectancies, the more they are likely to be cannabis users. Conversely, more negative effect expectancies are related to a lower probability of having tested cannabis. In the whole sample, negative expectancies demonstrated stronger correlations with lifetime cannabis use than positive expectancies. Among cannabis users, the frequency of use was also significantly positively correlated with positive expectancies and negatively with negative ones. Frequency of use increased when adolescents expected Perceptual Enhancement and Craving effects or Relaxation and Social Facilitation, and decreased when they expected negative effects of cannabis on their behavior. It is also noteworthy that the correlation between two factors, « Cognitive Impairment and Negative Effects » and « Negative Behavioral Effects », is quite high. This suggests that they could be merged into one unique factor related to the « negative effects » of cannabis use. Such a model improvement should be considered in future studies.

No significant relationship was found between the frequency of cannabis use and cognitive impairment expectancies, suggesting that, in this context of non-clinical users, the amount of cannabis use is not associated with expectancies related to cognitive damage. This result, which contrasts with Guillem et al.’s (2011) study, could be explained by the divergent methodologies, especially in terms of participants’ age (adults vs. adolescents), clinical status (psychiatric vs. non-clinical) and levels of substance use (dependent vs. young users).

Finally, the present study tested the predictive validity of the French MEEQ with a one-year follow-up. Specifically, adolescents who expected negative effects of cannabis consumption at T1 were less likely to have used cannabis at T2 and remained “non-users,” whereas those who expected positive effects were more likely to remain “users” or to become “new users” one year later. Note that expectancies concerning negative effects on behavior had the highest influence and resulted in the strongest probability of remaining non-users. These findings are in agreement with the results of Kristjansson et al. (2012) suggesting that reduced negative expectancies about the effects of cannabis can be a risk factor for using this substance. Moreover, concerning the prediction of frequency of use among cannabis users, only two specific expectancies were significant predictors: higher levels of « Perceptual Enhancement and Craving » expectancy predicted a greater frequency of use, whereas higher levels of « Negative Behavioral Effects » expectancy predicted a lower frequency of use. In contrast to its relationship with the prevalence of use (being a user vs. a non-user), the « Relaxation and Social Facilitation » expectancy did not significantly predict the frequency of use in cannabis users. This pattern of results suggests that the role of this latter expectancy changes from the stage of cannabis initiation (positive role) to the stage of maintaining or increasing the frequency of use (no significant effect in the present study). However, it is noteworthy the effect sizes obtained in the present study are relatively low (although significant) with small odd ratios. This indicates that the MEEQ is an useful, but not sufficient, instrument to discriminate adolescents who are at risk of using cannabis in the future from those who are not.

The main strengths of the present study are its large sample size and the inclusion of both cannabis users and non-users. All analyses were based on a previous exploratory study (Guillem et al., 2011), the results of which were confirmed by the present study. Moreover, the longitudinal design of the present study provided enough information to test the predictive validity, which is often missing in validation studies. The MEEQ scale itself is particularly interesting because it can be used with consumers and non-consumers of cannabis, expanding its practicality. Finally, the present results suggest that the shorter version of the original MEEQ, with 31 items and four factors, could be very practical to use, while it requires less time for administration. A shorter version of the scale (maybe without the less-significant items) might be considered in future studies for assessing the general population. However, although a validation in other settings is critical due to the influence of the social situation and the context (Piontek, Kraus, Bjarnason, Demetrovics, & Ramstedt, 2013), the present results with the four-factor structure should also be replicated in other populations (e.g. adult or clinical samples) and countries (e.g. English-speaking population) before such a scale can be recommended. In particular, clinical samples should be tested to generalize the use of this scale in clinical contexts. Then, the use of this questionnaire may be considered by health professionals in order to provide a more rigorous measure about cannabis effect expectancies. Prevention, risk reduction and therapeutic interventions might be focused on relevant expectancies and therefore be more adapted to the patient’s profile.

### Limitations and future studies

Several limitations of this study must be noted, providing guidance for future research.

First, the present study did not include a specific sample of clinical participants and is therefore not able to test differences between light and heavy cannabis users. Future studies should assess the French version of the MEEQ in teenagers who are cannabis abusers, cannabis dependent or have problems with the use of this substance. Such studies would provide information about the discriminant validity of the questionnaire. Another group of participants that cannot be unambiguously identified with a one-year follow-up study in young teenagers are cannabis quitters. Such a specific population of teenagers who have tried cannabis but will not maintain its consumption might have specific cannabis effect expectancies that would be relevant to study.

Second, the participants were only teenagers. It would be interesting to test the structural validity and psychometric properties of the French version of the MEEQ in an adult sample. Indeed, adolescence is a specific developmental period, quite distinct from childhood and adulthood, and is characterized by stressful events (Buchanan, Eccles, & Becker, 1992; Larson & Asmussen, 1991; Spear, 2007). Therefore, a validation in a sample of non-clinical adults (Guillem et al., 2011, used it in a clinical adults sample) is needed before using the questionnaire in such a population.

Third, the constructive validity was not assessed in the present study and must be confirmed by correlating this scale with other French scales of cannabis use expectancies. Moreover, the test-retest reliability was not assessed. Fourth, it would be useful to examine the association between responses on the French MEEQ and non-self-report measures (e.g. an adapted Implicit Association Test; Greenwald, McGhee & Schwartz, 1998). Finally, the respondents were only selected from the French-speaking part of Belgium, limiting the generalization of our finding to other French-speaking countries.

## Conclusion

To conclude, the French version of the MEEQ is an appropriate instrument to measure cannabis effect expectancies in adolescents, due to its factor structure and psychometric properties. According to Guillem et al. (2011), this scale describes four kinds of expectancies that may be considered separately: Cognitive Impairment and Negative Effects, Relaxation and Social Facilitation, Perceptual Enhancement and Craving, and Negative Behavioral Effects. The present results support the use of the French version of the MEEQ in an adolescent population. Data are available from the first author.

## Funding Statement

This work was supported by grants from the Fonds National de la Recherche Scientifique (FNRS). Emilie Schmits is a research assistant under contract with the FNRS.

## Competing Interest

The authors declare that they have no competing interests.

## Appendix A

### French Translation of the MEEQ

Les phrases suivantes contiennent des propositions à propos des effets du cannabis. **Si tu as déjà consommé du cannabis,** lis chaque question avec attention et réponds selon tes propres pensées, sentiments et croyances actuels à propos du cannabis, sans tenir compte de ce que les autres peuvent penser. **Si tu n’as jamais consommé du cannabis,** réponds en fonction de comment tu penses être affecté si tu en consommais. Réponds franchement. Il n’y a ni bonne ni mauvaise réponse. Entoure le chiffre qui correspond à quel point tu es d’accord ou pas d’accord avec chaque proposition.

**Table:**

1	2	3	4	5

Pas du tout d’accord	Plutôt pas d’accord	Ne sais pas	Plutôt d’accord	Tout à fait d’accord

**Table:**

1.	Le cannabis rend les petites choses intensément intéressantes.	1	2	3	4	5
2.	Fumer du cannabis me donne faim.	1	2	3	4	5
3.	Fumer du cannabis augmente mon désir immédiat des choses.	1	2	3	4	5
4.	Je trouve une sensation de relaxation en fumant du cannabis.	1	2	3	4	5
5.	Fumer du cannabis me rend moins tendu(e) ou soulage mon anxiété, il m’aide à me décontracter.	1	2	3	4	5
6.	Le cannabis me rend insouciant(e) et je me moque de mes problèmes.	1	2	3	4	5
7.	Je ne suis pas préoccupé(e) par la manière dont les autres me jugent quand je suis sous cannabis.	1	2	3	4	5
8.	Le cannabis me fait parler plus que d’habitude.	1	2	3	4	5
9.	J’ai l’impression que je peux mieux me focaliser sur une chose particulière quand je fume du cannabis.	1	2	3	4	5
10.	Quand je fume du cannabis, je ne me sens pas angoissé.	1	2	3	4	5
11.	Le cannabis me fait dire des choses que je ne pense pas.	1	2	3	4	5
12.	Je suis plus sociable quand je fume du cannabis.	1	2	3	4	5
13.	Fumer du cannabis me donne l’impression que j’appartiens au groupe.	1	2	3	4	5
14.	Si j’ai fumé du cannabis, il est plus dur pour moi de me concentrer et de comprendre ce qui est dit.	1	2	3	4	5
15.	Le cannabis ralentit mes pensées et mes actions	1	2	3	4	5
16.	Je deviens plus créatif(ve) ou imaginatif(ve) sous cannabis.	1	2	3	4	5
17.	Si j’ai fumé du cannabis il est plus dur de me souvenir des choses.	1	2	3	4	5
18.	Les choses semblent irréelles et je me sens déconnecté(e) de ce qui se passe autour de moi quand je fume du cannabis.	1	2	3	4	5
19.	Quand je fume du cannabis, cela m’aide à échapper à la réalité.	1	2	3	4	5
20.	Le cannabis me fait rire pour un rien et rire beaucoup.	1	2	3	4	5
21.	Quand je fume du cannabis j’ai l’impression d’avoir les pieds lourds et de manquer de coordination.	1	2	3	4	5
22.	La musique a un son différent quand je fume du cannabis.	1	2	3	4	5
23.	Le cannabis a un mauvais goût et une mauvaise odeur.	1	2	3	4	5
24.	J’ai un sentiment heureux et agréable quand je fume du cannabis.	1	2	3	4	5
25.	Le cannabis me fait perdre le contrôle et devenir négligent.	1	2	3	4	5
26.	Le cannabis permet d’échapper plus facilement aux problèmes et aux responsabilités.	1	2	3	4	5
27.	Je suis moins motivé(e) quand je fume du cannabis.	1	2	3	4	5
28.	Le cannabis me rend déprimé(e) et déçu(e) de moi-même.	1	2	3	4	5
29.	Le cannabis provoque de l’euphorie (forte sensation de bien-être).	1	2	3	4	5
30.	Le cannabis peut faire passer mes sentiments de la joie à la tristesse.	1	2	3	4	5
31.	J’agis de façon excitée quand je fume du cannabis.	1	2	3	4	5
32.	Le cannabis ne me rend pas plus romantique, ni plus attiré(e) par d’autres personnes.	1	2	3	4	5
33.	Après avoir fumé du cannabis mes paupières deviennent lourdes et je deviens somnolent(e).	1	2	3	4	5
34.	Le cannabis peut me mettre en colère et me rend potentiellement violent(e).	1	2	3	4	5
35.	Une fois que je me suis défoncé(e) en fumant du cannabis, je n’ai pas le moral.	1	2	3	4	5
36.	Le cannabis n’altère pas ma personnalité.	1	2	3	4	5
37.	me sens attirant(e) ou plus intéressé(e) par le sexe après avoir fumé du cannabis.	1	2	3	4	5
38.	Le cannabis me rend critique et d’humeur irritable.	1	2	3	4	5
39.	« J’ai les crocs » (envie de casser la croûte) quand je fume du cannabis.	1	2	3	4	5
40.	Il est difficile pour moi d’exprimer clairement mes pensées si j’ai fumé du cannabis.	1	2	3	4	5
41.	Le cannabis me donne l’impression d’avoir la bouche sèche.	1	2	3	4	5
42.	Le cannabis me rend calme.	1	2	3	4	5
43.	Le cannabis change ma perception du temps et des distances.	1	2	3	4	5
44.	Je suis plus détendu(e) dans les situations sociales si j’ai fumé du cannabis.	1	2	3	4	5
45.	Je m’amuse mieux dans les soirées si je fume du cannabis	1	2	3	4	5
46.	Le cannabis ralentit mes réactions.	1	2	3	4	5
47.	Je suis plus partant pour faire les choses que normalement je ne ferais pas quand je fume du cannabis.	1	2	3	4	5
48.	Fumer du cannabis, c’est presque comme être saoul avec de l’alcool.	1	2	3	4	5

*Note*. The shaded items are excluded from the French validation with the four-factor structure (but are present in the complete English six-factor structure).

## Appendix B

### Validation data for the six-factor structure model

**Table 1:** Factor structure and internal consistency reliability.

	X^2^ (dl)	CFI	RMSEA	Factor loadings	α

CBI	84.67 (35)	.95	.03	>.40 excepted items 36 and 46	.79
RTR	136.08 (20)	.92	.07	>.40 excepted item 26	.81
SSF	112.89 (27)	.77	.05	>.40 excepted items 8 and 12	.56
PCE	119.42 (20)	.85	.06	>.40 excepted items 9, 22 and 37	.67
GNE	187.30 (27)	.88	.07	>.40 excepted items 30, 35 and 48	.80
CPE	70.87 (9)	.85	.07	>.40 excepted items 33	.66

*Note*. CBI = Cognitive and Behavioral Impairments (items 14, 15, 17, 18, 21, 33, 36, 40, 43, 46), RTR = Relaxation and Tension Reduction (items 4, 5, 6, 7, 10, 26, 42, 44), SSF = Social and Sexual Facilitation (items 8, 12, 13, 24, 27, 31, 32, 45, 47), PCE = Perceptual and Cognitive Enhancement (items 1, 9, 16, 19, 22, 26, 29, 37), GNE = Global Negative Effects (items 11, 23, 25, 28, 30, 34, 35, 38, 48), CPE = Craving and Physical Effects (items 2, 3, 20, 33, 39, 41).

**Table 2:** Criterion Validity.

	Whole sample (n = 1343)							Users (n = 325)
	
	1.	2.	3.	4.	5.	6.	7.	8.

1. Lifetime cannabis	1.00							
2. CBI	–0.13**	1.00						
3. RTR	0.18**	0.53**	1.00					
4. SSF	–.01	0.54**	0.64**	1.00				
5. PCE	.01	0.60**	0.77**	0.70**	1.00			
6. GNE	–.49**	.62**	.24**	.42**	.43**	1.00		
7. CPE	.20**	.61**	.67**	.58**	.68**	.33**	1.00	
8. Frequency of use		0.11*	.28**	0.07	.32**	–.26**	.34**	1.00

Note. CBI = Cognitive and Behavioral Impairments, RTR = Relaxation and Tension Reduction, SSF = Social and Sexual Facilitation, PCE = Perceptual and Cognitive Enhancement, GNE = Global Negative Effects, CPE = Craving and Physical Effects. **p* < 0.05, ***p* < 0.001.

**Table 3:** Predictive Validity Results of the Multinomial Logistic Regressions Analyses of Profiles of Cannabis Users.

	b	SE b	Wald	OR (95% CI)

Users at baseline vs. Non-users				
Constant	–3.65**	0.91	16.17	
CBI	0.01	0.03	0.21	1.01 (0.95–1.07)
RTR	–0.001	0.03	0.001	0.99 (0.93–1.06)
SSF	0.07*	0.03	5.27	1.07 (1.01–1.14)
PCE	0.03	0.04	0.64	1.03 (0.95–1.12)
GNE	–0.12**	.03	18.53	0.88 (0.83–0.93)
CPE	0.11*	0.4	6.08	1.12 (1.02–1.22)
New users vs. Non-users				
Constant	–0.28	0.53	0.29	
CBI	–0.04	0.03	1.86	0.96 (0.91–1.01)
RTR	0.07*	0.03	4.74	1.07 (1.01–1.14)
SSF	0.02	0.03	0.77	1.02 (.96–1.09)
PCE	–0.01	0.04	0.02	0.99 (0.91–1.07)
GNE	–0.34**	0.03	139.81	0.71 (0.67–0.75)
CPE	0.32**	0.04	57.52	1.38 (1.27–1.50)

*Note*. Non-users was the reference category. OR = Odds Ratio. CBI = Cognitive and Behavioral Impairments, RTR = Relaxation and Tension Reduction, SSF = Social and Sexual Facilitation, PCE = Perceptual and Cognitive Enhancement, GNE = Global Negative Effects, CPE = Craving and Physical Effects. **p*< 0.05, ***p*< 0.001.

**Table 4:** Predictive Validity – Results of the Multiple Regression Analyses of Usual Frequency of Cannabis Use.

	b	SE b	ß

MEEQ			
Constant	2.93 (1.94, 3.92)**	0.50	
CBI	0.01 (–0.03, 0.03)	0.02	0.02
RTR	–0.04 (–0.08, –0.01)*	0.02	–0.18
SSF	0.01 (–0.02, 0.04)	0.02	0.04
PCE	0.03 (–0.01, 0.07)	0.02	0.11
GNE	–0.08 (–0.11, –0.06)**	0.01	–0.43
CPE	0.07 (0.02, 0.11)*	0.02	0.21

*Note*. Multiple regression with 95% bias corrected and accelerated confidence intervals reported in parentheses. Confidence intervals and standard errors based on 1000 bootstrap samples. CBI = Cognitive and Behavioral Impairments, RTR = Relaxation and Tension Reduction, SSF = Social and Sexual Facilitation, PCE = Perceptual and Cognitive Enhancement, GNE = Global Negative Effects, CPE = Craving and Physical Effects. **p* < 0.05, ***p* < 0.001.
